# 1,3,5-Tris(pyridin-3-yl)-2,4-diaza­penta-1,4-diene

**DOI:** 10.1107/S1600536812005909

**Published:** 2012-02-17

**Authors:** Claudia M. Quiroa-Montalván, Gerardo Aguirre, Miguel Parra-Hake

**Affiliations:** a Centro de Graduados e Investigación del Instituto Tecnológico de Tijuana, Apdo. Postal 1166, 22500, Tijuana, B.C., Mexico

## Abstract

In the solid state, the structure of the title compound, C_18_H_15_N_5_, is stabilized by weak C—H⋯N interactions. Mol­ecules are arranged in layers parallel to the *bc* plane forming an inter­esting supra­molecular structure.

## Related literature

For coordination polymers and supra­molecular structures, see: Itoh *et al.* (2005 ▶); Albrechet (2001 ▶); Leininger *et al.* (2000 ▶). For potential applications in catalysis, gas storage, chirality, optics, magnetism, nanotechnology and luminescence, see: James (2003 ▶); Kitagawa *et al.* (2004 ▶); Masaoka *et al.* (2001 ▶); Rarig *et al.* (2002 ▶); Yaghi *et al.* (2003 ▶); Wang *et al.* (2009 ▶). For the preparation of this class of compound, see: Larter *et al.* (1998 ▶); Lozinskaya *et al.* (2003 ▶); Bessonov *et al.* (2005 ▶); Fernandes *et al.* (2007 ▶).
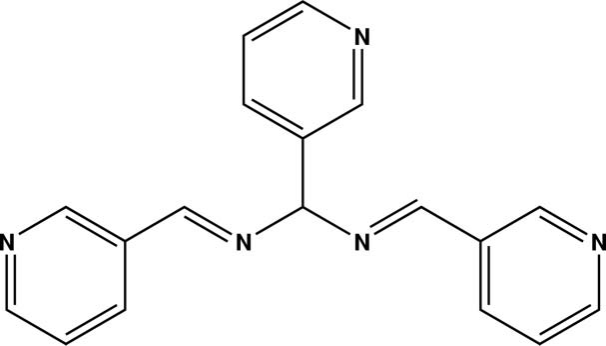


## Experimental

### 

#### Crystal data

C_18_H_15_N_5_
*M* *_r_* = 301.35
Monoclinic, 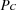

*a* = 5.7174 (11) Å
*b* = 8.0934 (10) Å
*c* = 16.972 (4) Å
β = 99.690 (18)°
*V* = 774.1 (3) Å^3^
*Z* = 2
Mo *K*α radiation
μ = 0.08 mm^−1^
*T* = 298 K
0.42 × 0.18 × 0.12 mm

#### Data collection

Bruker P4 diffractometer
3235 measured reflections
2874 independent reflections
1159 reflections with *I* > 2σ(*I*)
*R*_int_ = 0.061
3 standard reflections every 97 reflections intensity decay: 11.5%

#### Refinement

*R*[*F* ^2^ > 2σ(*F* ^2^)] = 0.070
*wR*(*F* ^2^) = 0.135
*S* = 0.98
2874 reflections
209 parameters
2 restraints
H-atom parameters constrained
Δρ_max_ = 0.17 e Å^−3^
Δρ_min_ = −0.17 e Å^−3^

### 

Data collection: *XSCANS* (Siemens, 1996 ▶); cell refinement: *XSCANS*; data reduction: *XSCANS*; program(s) used to solve structure: *SHELXS97* (Sheldrick, 2008 ▶); program(s) used to refine structure: *SHELXL97* (Sheldrick, 2008 ▶); molecular graphics: *ORTEP-3* (Farrugia, 1997 ▶) and *Mercury* (Macrae *et al.*, 2006 ▶); software used to prepare material for publication: *SHELXS97*.

## Supplementary Material

Crystal structure: contains datablock(s) I, global. DOI: 10.1107/S1600536812005909/rk2325sup1.cif

Structure factors: contains datablock(s) I. DOI: 10.1107/S1600536812005909/rk2325Isup2.hkl

Supplementary material file. DOI: 10.1107/S1600536812005909/rk2325Isup3.cml

Additional supplementary materials:  crystallographic information; 3D view; checkCIF report

## Figures and Tables

**Table 1:** Hydrogen-bond geometry (Å, °)

*D*—H⋯*A*	*D*—H	H⋯*A*	*D*⋯*A*	*D*—H⋯*A*
C18—H18*A*⋯N1^i^	0.93	2.74	3.552 (7)	146
C17—H17*A*⋯N3^ii^	0.93	2.66	3.456 (7)	144

Symmetry codes: (i) 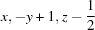; (ii) 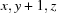.

**Fig. 1.**
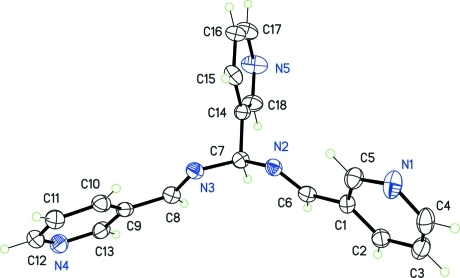
Molecular structure of title compound with the atom numbering scheme. The displacement ellipsoids are drawn at 30% probability level. H atoms are presented as a small spheres of arbitrary radius.

**Fig. 2.**
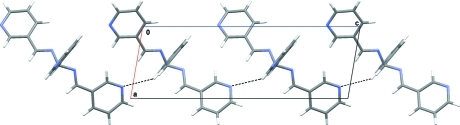
The diagram showing the H-bonds. The molecules are forming an array along the [0 0 1] direction. H-bonds are indicated by broken lines.

## supplementary crystallographic information

### Comment

The coordination chemistry of transition metals with polypyridyl ligands has progressed considerably during the last decades, and has been widely used for the construction of coordination polymers and other supramolecular structures (Itoh *et al.*, 2005; Albrechet, 2001; Leininger *et al.*, 2000). Such supramolecular architectures have attracted considerable attention due to potential applications in catalysis, gas storage, chirality, optical, magnetism, nanotechnology and luminescence (James, 2003; Kitagawa *et al.*, 2004; Masaoka *et al.*, 2001; Rarig *et al.*, 2002; Yaghi *et al.*, 2003; Wang *et al.*, 2009). Also, there exists an increasing interest in the design and synthesis of luminescent compounds due to their potential applications as chemical sensors, photochemistry and electroluminescence. The development of chemosensors, is one of the main goals of supramolecular chemistry and an important area of vigorous investigation.

We are interested on the coordination chemistry of polypyridine ligands, which have fluorescent properties and could act as sensors for transition metals ions, and which can be used to construct different coordination polymers. Some of the ligands under study are: *cis*-(±)-2,4,5-tri(2-pyridyl)imidazoline, 2,4,6-tri(2-pyridyl)-1,3,5-triazinane, 2,4,5-tri(2-pyridyl)imidazole, *trans*-(±)-2,4,5-tri(4-pyridyl)imidazoline and 2,4,5-tri(4-pyridyl)imidazole.

As part of our ongoing research on the chemistry of polypyridine ligands, in our attempts to synthesize the ligand *cis*-(±)-3-(2,5-di(pyridin-3-yl)-4,5-dihydro-1*H*-imidazol-4-yl) pyridine, we have isolated the title compound, 1,3,5-tri(pyridin-3-yl)-2,4-diazapenta-1,4-diene. The 1,3,5-triaryl-2,4-diazapentadienes are known to form by the reaction of aromatic benzaldehydes with ammonia (Larter *et al.*, 1998; Lozinskaya *et al.*, 2003; Bessonov *et al.*, 2005; Fernandes *et al.*, 2007), which are analogues of the title compound. In the crystal structure adjacent networks are linked together *via* intermolecular hydrogen bond interactions (Table 1) (C18–H18···N1^i^ (2.741Å), symmetry code: (i) *x*, 1-*y*, -1/2+*z*) in an array along the [0 0 1] and [C17–H17···N3^ii^ (2.660Å), symmetry codes: (ii) *x*, 1+*y*, *z*] in an array along the [0 1 0]. The molecules are forming a layer structure parallel to the *bc* plane (Fig. 2).

### Experimental

The synthesis of the title compound included reagent grade starting materials and solvents. A mixture of pyridine-3-carboxaldehyde (5 mL, 0.0531 mol) and ammonium hydroxide (15 mL, 0.3843 mol) was stirred at room temperature for 24 h. The mixture was filtered off and washed with water, then recrystallized by gas phase diffusion of diethyl ether into a concentrated solution of the product in dichloromethane, providing colorless crystals. Yield (3.5 g, 22%). M.p. = 388-390 K, (KBr) 3270, 3226, 3040, 2887, 1575, 1471, 1417, 1082, 1023, 868, 863, 808, 705 cm^-1^. ^1^H NMR (CDCl_3_): 8.98 (d, *J* = 1.8 Hz, 2H), 8.79 (d, *J* = 1.8 Hz, 1H), 8.69 (dd, *J* = 4.8, 1.8 Hz, 2H), 8.66 (s, 2H), 8.59 (dd, *J* = 4.8, 2.1 Hz, 1H), 8.26 (ddd, *J* = 7.8, 1.8, 1.8 Hz, 2H), 7.87 (ddd, *J* = 8.2, 2.1, 1.8 Hz, 1H), 7.39 (dd, *J* = 7.8, 4.8 Hz, 2H), 7.33 (dd, *J* = 8.2, 4.8 Hz, 1H), 6.08 (s, 1H). ^13^C NMR (CDCl_3_): 158.94, 152.21, 150.75, 149.53, 148.89, 136.61, 134.98, 134.83, 131.01, 123,73, 123.64, 90.28. EIMS (70 eV) m/e (int. *rel.*): *M*^+^ 301 (1%), *M*^+^-*Py*-CH═N 196 (100%), 168 (13%), 122 (3%), 92 (10%).

### Refinement

Refinement for H atoms was carried out using a riding model, with distances constrained to: 0.93Å for aromatic C–H, 0.98Å for methine C–H with *U*_iso_(H) = 1.2*U*_eq_(C). The 621 Friedel pairs were merged during refinement.

### Crystal data

**Table:**

C_18_H_15_N_5_	*F*(000) = 316
*M**_r_* = 301.35	*D*_x_ = 1.293 Mg m^−^^3^
Monoclinic, *P**c*	Melting point = 388–390 K
Hall symbol: P -2yc	Mo *K*α radiation, λ = 0.71073 Å
*a* = 5.7174 (11) Å	Cell parameters from 33 reflections
*b* = 8.0934 (10) Å	θ = 4.7–11.6°
*c* = 16.972 (4) Å	µ = 0.08 mm^−^^1^
β = 99.690 (18)°	*T* = 298 K
*V* = 774.1 (3) Å^3^	Neele, colourless
*Z* = 2	0.42 × 0.18 × 0.12 mm

### Data collection

**Table:**

Bruker P4 diffractometer	*R*_int_ = 0.061
Radiation source: fine-focus sealed tube	θ_max_ = 30.0°, θ_min_ = 2.4°
Graphite monochromator	*h* = −1→8
2θ/ω–scans	*k* = −1→11
3235 measured reflections	*l* = −23→23
2874 independent reflections	3 standard reflections every 97 reflections
1159 reflections with *I* > 2σ(*I*)	intensity decay: 11.5%

### Refinement

**Table:**

Refinement on *F*^2^	Hydrogen site location: inferred from neighbouring sites
Least-squares matrix: full	H-atom parameters constrained
*R*[*F*^2^ > 2σ(*F*^2^)] = 0.070	*w* = 1/[σ^2^(*F*_o_^2^) + (0.0352*P*)^2^] where *P* = (*F*_o_^2^ + 2*F*_c_^2^)/3
*wR*(*F*^2^) = 0.135	(Δ/σ)_max_ < 0.001
*S* = 0.98	Δρ_max_ = 0.17 e Å^−^^3^
2874 reflections	Δρ_min_ = −0.17 e Å^−^^3^
209 parameters	Extinction correction: *SHELXL*, Fc^*^=kFc[1+0.001xFc^2^λ^3^/sin(2θ)]^-1/4^
2 restraints	Extinction coefficient: 0.019 (2)
Primary atom site location: structure-invariant direct methods	Absolute structure: Flack (1983)
Secondary atom site location: difference Fourier map	

### Special details

**Table:**

Geometry. All s.u.'s (except the s.u. in the dihedral angle between two l.s. planes) are estimated using the full covariance matrix. The cell s.u.'s are taken into account individually in the estimation of s.u.'s in distances, angles and torsion angles; correlations between s.u.'s in cell parameters are only used when they are defined by crystal symmetry. An approximate (isotropic) treatment of cell s.u.'s is used for estimating s.u.'s involving l.s. planes.
Refinement. Refinement of *F*^2^ against ALL reflections. The weighted *R*-factor *wR* and goodness of fit *S* are based on *F*^2^, conventional *R*-factors *R* are based on *F*, with *F* set to zero for negative *F*^2^. The threshold expression of *F*^2^ > σ(*F*^2^) is used only for calculating *R*-factors(gt) *etc*. and is not relevant to the choice of reflections for refinement. *R*-factors based on *F*^2^ are statistically about twice as large as those based on *F*, and *R*-factors based on ALL data will be even larger.

### Fractional atomic coordinates and isotropic or equivalent isotropic displacement parameters (Å^2^)

**Table:**

	*x*	*y*	*z*	*U*_iso_*/*U*_eq_	
N3	0.3294 (7)	0.4546 (5)	0.0999 (2)	0.0434 (11)	
C4	1.0463 (12)	0.2145 (8)	0.4651 (3)	0.0578 (18)	
H4A	1.0947	0.1666	0.5150	0.069*	
N2	0.6027 (8)	0.4678 (5)	0.2201 (2)	0.0408 (11)	
C3	1.1993 (11)	0.2058 (7)	0.4105 (3)	0.0588 (18)	
H3B	1.3474	0.1561	0.4235	0.071*	
C7	0.5371 (9)	0.5446 (6)	0.1421 (3)	0.0413 (14)	
H7A	0.6689	0.5367	0.1120	0.050*	
C8	0.3369 (10)	0.4143 (6)	0.0281 (3)	0.0436 (14)	
H8A	0.4684	0.4440	0.0057	0.052*	
C16	0.2483 (11)	0.9366 (7)	0.1982 (3)	0.0486 (15)	
H16A	0.1265	0.9714	0.2243	0.058*	
N4	−0.0186 (9)	0.2211 (7)	−0.1527 (2)	0.0604 (15)	
C2	1.1242 (10)	0.2739 (7)	0.3358 (3)	0.0481 (15)	
H2B	1.2222	0.2715	0.2973	0.058*	
C13	0.1481 (10)	0.2988 (7)	−0.1022 (3)	0.0497 (16)	
H13A	0.2775	0.3410	−0.1223	0.060*	
C14	0.4741 (10)	0.7230 (6)	0.1524 (3)	0.0402 (13)	
C1	0.9022 (9)	0.3453 (7)	0.3190 (3)	0.0404 (14)	
C12	−0.1994 (11)	0.1565 (7)	−0.1222 (3)	0.0587 (18)	
H12A	−0.3174	0.1006	−0.1564	0.070*	
C6	0.8136 (10)	0.4183 (7)	0.2402 (3)	0.0443 (15)	
H6A	0.9172	0.4283	0.2037	0.053*	
N1	0.8360 (8)	0.2859 (7)	0.4515 (2)	0.0638 (16)	
C9	0.1421 (10)	0.3216 (7)	−0.0208 (3)	0.0411 (14)	
C11	−0.2194 (10)	0.1688 (7)	−0.0427 (3)	0.0555 (17)	
H11A	−0.3465	0.1201	−0.0238	0.067*	
C10	−0.0493 (10)	0.2540 (7)	0.0083 (3)	0.0476 (15)	
H10A	−0.0625	0.2662	0.0619	0.057*	
C15	0.2938 (10)	0.7702 (7)	0.1923 (3)	0.0482 (15)	
H15A	0.2052	0.6919	0.2145	0.058*	
C18	0.5982 (12)	0.8483 (7)	0.1210 (3)	0.0558 (16)	
H18A	0.7196	0.8174	0.0938	0.067*	
C5	0.7697 (10)	0.3477 (7)	0.3793 (3)	0.0495 (15)	
H5A	0.6207	0.3969	0.3683	0.059*	
C17	0.3808 (11)	1.0503 (8)	0.1661 (3)	0.0627 (18)	
H17A	0.3474	1.1617	0.1718	0.075*	
N5	0.5548 (11)	1.0099 (6)	0.1272 (3)	0.0731 (18)	

### Atomic displacement parameters (Å^2^)

**Table:**

	*U*^11^	*U*^22^	*U*^33^	*U*^12^	*U*^13^	*U*^23^
N3	0.051 (3)	0.040 (3)	0.039 (2)	0.001 (3)	0.008 (2)	−0.001 (2)
C4	0.063 (5)	0.062 (5)	0.042 (3)	−0.010 (4)	−0.008 (3)	0.019 (3)
N2	0.050 (3)	0.039 (3)	0.033 (2)	0.000 (3)	0.005 (2)	0.001 (2)
C3	0.046 (4)	0.055 (4)	0.069 (4)	0.001 (4)	−0.008 (3)	0.007 (3)
C7	0.043 (4)	0.044 (3)	0.038 (3)	0.002 (3)	0.010 (3)	0.002 (3)
C8	0.049 (4)	0.041 (3)	0.041 (3)	−0.001 (3)	0.010 (3)	0.003 (3)
C16	0.060 (4)	0.041 (4)	0.049 (3)	0.002 (4)	0.019 (3)	−0.006 (3)
N4	0.061 (4)	0.073 (4)	0.044 (3)	0.011 (4)	0.000 (3)	−0.013 (3)
C2	0.042 (4)	0.057 (4)	0.046 (3)	0.002 (4)	0.010 (3)	−0.006 (3)
C13	0.050 (4)	0.060 (4)	0.038 (3)	0.014 (4)	0.006 (3)	0.000 (3)
C14	0.048 (4)	0.040 (3)	0.032 (3)	0.006 (4)	0.004 (3)	0.004 (3)
C1	0.039 (4)	0.043 (3)	0.037 (3)	−0.003 (3)	0.002 (3)	0.001 (3)
C12	0.059 (5)	0.054 (4)	0.057 (4)	0.006 (4)	−0.006 (3)	−0.016 (3)
C6	0.050 (4)	0.042 (3)	0.043 (3)	0.002 (3)	0.012 (3)	−0.005 (3)
N1	0.050 (4)	0.089 (4)	0.049 (3)	−0.004 (4)	−0.001 (3)	0.015 (3)
C9	0.048 (4)	0.037 (3)	0.038 (3)	0.009 (3)	0.007 (3)	−0.001 (3)
C11	0.051 (4)	0.057 (4)	0.058 (4)	0.002 (4)	0.009 (3)	−0.009 (3)
C10	0.057 (4)	0.047 (4)	0.039 (3)	0.008 (4)	0.007 (3)	−0.003 (3)
C15	0.056 (4)	0.048 (4)	0.042 (3)	−0.017 (4)	0.014 (3)	−0.002 (3)
C18	0.058 (4)	0.054 (4)	0.061 (3)	−0.001 (4)	0.028 (3)	−0.005 (3)
C5	0.041 (4)	0.059 (4)	0.048 (3)	0.000 (4)	0.007 (3)	0.004 (3)
C17	0.089 (6)	0.043 (4)	0.061 (3)	−0.001 (4)	0.026 (4)	−0.002 (3)
N5	0.101 (5)	0.050 (4)	0.080 (3)	−0.001 (4)	0.051 (4)	0.010 (3)

### Geometric parameters (Å, º)

**Table:**

N3—C8	1.269 (5)	C13—C9	1.401 (6)
N3—C7	1.472 (6)	C13—H13A	0.9300
C4—N1	1.319 (7)	C14—C15	1.378 (7)
C4—C3	1.380 (8)	C14—C18	1.394 (7)
C4—H4A	0.9300	C1—C5	1.372 (7)
N2—C6	1.262 (6)	C1—C6	1.473 (7)
N2—C7	1.454 (6)	C12—C11	1.376 (7)
C3—C2	1.383 (7)	C12—H12A	0.9300
C3—H3B	0.9300	C6—H6A	0.9300
C7—C14	1.506 (6)	N1—C5	1.319 (6)
C7—H7A	0.9800	C9—C10	1.387 (7)
C8—C9	1.476 (7)	C11—C10	1.374 (7)
C8—H8A	0.9300	C11—H11A	0.9300
C16—C17	1.363 (7)	C10—H10A	0.9300
C16—C15	1.378 (7)	C15—H15A	0.9300
C16—H16A	0.9300	C18—N5	1.339 (7)
N4—C13	1.327 (7)	C18—H18A	0.9300
N4—C12	1.338 (8)	C5—H5A	0.9300
C2—C1	1.380 (7)	C17—N5	1.324 (7)
C2—H2B	0.9300	C17—H17A	0.9300
			
C8—N3—C7	116.0 (4)	C5—C1—C6	121.5 (5)
N1—C4—C3	124.5 (5)	C2—C1—C6	121.3 (5)
N1—C4—H4A	117.7	N4—C12—C11	123.2 (6)
C3—C4—H4A	117.7	N4—C12—H12A	118.4
C6—N2—C7	118.0 (4)	C11—C12—H12A	118.4
C4—C3—C2	117.5 (6)	N2—C6—C1	122.6 (5)
C4—C3—H3B	121.2	N2—C6—H6A	118.7
C2—C3—H3B	121.2	C1—C6—H6A	118.7
N2—C7—N3	107.1 (4)	C5—N1—C4	116.1 (5)
N2—C7—C14	109.5 (4)	C10—C9—C13	116.8 (5)
N3—C7—C14	110.0 (4)	C10—C9—C8	124.5 (5)
N2—C7—H7A	110.1	C13—C9—C8	118.7 (5)
N3—C7—H7A	110.1	C12—C11—C10	119.2 (6)
C14—C7—H7A	110.1	C12—C11—H11A	120.4
N3—C8—C9	121.7 (5)	C10—C11—H11A	120.4
N3—C8—H8A	119.1	C11—C10—C9	119.4 (5)
C9—C8—H8A	119.1	C11—C10—H10A	120.3
C17—C16—C15	120.4 (6)	C9—C10—H10A	120.3
C17—C16—H16A	119.8	C16—C15—C14	118.2 (5)
C15—C16—H16A	119.8	C16—C15—H15A	120.9
C13—N4—C12	117.0 (5)	C14—C15—H15A	120.9
C1—C2—C3	119.2 (5)	N5—C18—C14	124.5 (6)
C1—C2—H2B	120.4	N5—C18—H18A	117.7
C3—C2—H2B	120.4	C14—C18—H18A	117.7
N4—C13—C9	124.4 (5)	N1—C5—C1	125.4 (6)
N4—C13—H13A	117.8	N1—C5—H5A	117.3
C9—C13—H13A	117.8	C1—C5—H5A	117.3
C15—C14—C18	117.2 (5)	N5—C17—C16	123.2 (6)
C15—C14—C7	122.4 (5)	N5—C17—H17A	118.4
C18—C14—C7	120.3 (5)	C16—C17—H17A	118.4
C5—C1—C2	117.2 (5)	C17—N5—C18	116.4 (6)
			
N1—C4—C3—C2	−1.4 (9)	N4—C13—C9—C10	1.6 (8)
C6—N2—C7—N3	125.7 (5)	N4—C13—C9—C8	−178.4 (5)
C6—N2—C7—C14	−115.1 (5)	N3—C8—C9—C10	−7.8 (8)
C8—N3—C7—N2	−133.5 (5)	N3—C8—C9—C13	172.3 (5)
C8—N3—C7—C14	107.6 (5)	N4—C12—C11—C10	1.3 (9)
C7—N3—C8—C9	179.1 (4)	C12—C11—C10—C9	−1.8 (8)
C4—C3—C2—C1	−0.5 (8)	C13—C9—C10—C11	0.4 (7)
C12—N4—C13—C9	−2.2 (8)	C8—C9—C10—C11	−179.5 (5)
N2—C7—C14—C15	−59.2 (6)	C17—C16—C15—C14	−0.7 (8)
N3—C7—C14—C15	58.3 (6)	C18—C14—C15—C16	0.1 (7)
N2—C7—C14—C18	121.2 (5)	C7—C14—C15—C16	−179.6 (5)
N3—C7—C14—C18	−121.4 (5)	C15—C14—C18—N5	0.3 (9)
C3—C2—C1—C5	1.5 (8)	C7—C14—C18—N5	180.0 (6)
C3—C2—C1—C6	−179.6 (5)	C4—N1—C5—C1	−1.1 (9)
C13—N4—C12—C11	0.6 (9)	C2—C1—C5—N1	−0.7 (9)
C7—N2—C6—C1	177.3 (4)	C6—C1—C5—N1	−179.7 (6)
C5—C1—C6—N2	−9.1 (8)	C15—C16—C17—N5	0.9 (9)
C2—C1—C6—N2	171.9 (6)	C16—C17—N5—C18	−0.5 (9)
C3—C4—N1—C5	2.2 (9)	C14—C18—N5—C17	−0.1 (9)

### Hydrogen-bond geometry (Å, º)

**Table:**

*D*—H···*A*	*D*—H	H···*A*	*D*···*A*	*D*—H···*A*
C18—H18*A*···N1^i^	0.93	2.74	3.552 (7)	146
C17—H17*A*···N3^ii^	0.93	2.66	3.456 (7)	144

Symmetry codes: (i) *x*, −*y*+1, *z*−1/2; (ii) *x*, *y*+1, *z*.
